# Emotion regulation mediates the relationship between family caregivers’ pain-related beliefs and patients’ coping strategies

**DOI:** 10.3389/fnbeh.2023.983350

**Published:** 2023-02-07

**Authors:** Fatemeh Alinajimi, Zoha Deldar, Mohsen Dehghani, Ali Khatibi

**Affiliations:** ^1^Family Research Institute, Shahid Beheshti University, Tehran, Iran; ^2^Department of Psychology, McGill University, Montreal, QC, Canada; ^3^Department of Anatomy, Université du Québec à Trois-Rivières, Trois-Rivières, QC, Canada; ^4^Department of Clinical Psychology and Health, Shahid Beheshti University, Tehran, Iran; ^5^Centre of Precision Rehabilitation for Spinal Pain, School of Sport, Exercise and Rehabilitation Sciences, College of Life and Environmental Sciences, University of Birmingham, Birmingham, United Kingdom; ^6^Centre for Human Brain Health, University of Birmingham, Birmingham, United Kingdom; ^7^Institute for Mental Health, University of Birmingham, Birmingham, United Kingdom

**Keywords:** chronic pain, pain coping strategies, emotion regulation (ER), chronic musculoskeletal pain (CMP), pain attitudes-beliefs, family caregiver

## Abstract

**Background:** In order to tailor more effective interventions and minimize the burden of chronic pain, it is critical to identify the interaction and contribution of social and psychological factors in pain. One of the important psychological factors in pain management is related to the choice of pain coping strategies in chronic pain patients. Social resources, including family caregivers’ pain attitudes-beliefs, can influence pain coping strategies in chronic pain patients. Moreover, one key factor that may intervene in the relationship between caregivers’ pain attitudes-beliefs and the patients’ coping strategies is the emotion regulation strategies. Therefore, the present study aimed to investigate the mediating role of emotion regulation strategies of chronic pain patients and their family caregivers on the association between caregivers’ pain attitudes-beliefs and pain coping strategies of chronic pain patients.

**Methods:** We recruited 200 chronic musculoskeletal pain patients and their family caregivers. Chronic pain patients responded to measures of pain coping and emotion regulation strategies while family caregivers completed questionnaires related to their attitude toward pain and emotion regulation of themselves.

**Results:** There is an association between caregivers’ pain attitudes-beliefs and pain coping strategies in patients with chronic musculoskeletal. Moreover, the structural equation modeling revealed that the emotion regulation of both patients and family caregivers mediate the relationship between the caregivers’ pain attitudes-beliefs and pain coping strategies of patients with chronic musculoskeletal.

**Conclusions:** The social context of pain, including the effect of family caregivers’ responses to the patient’s pain, is a critical pain source that is suggested to affect coping strategies in patients. These findings suggest an association between pain attitudes-beliefs in family caregivers and pain coping strategies in patients. Moreover, these results showed that the emotion regulation of both patients and their family caregivers mediates this association.

## 1. Introduction

Chronic pain is a complex and subjective experience influenced by biopsychosocial factors which makes pain management for chronic pain patients (CPPs) challenging (Osborne et al., 2007; Ferreira-Valente et al., 2014; Edwards et al., 2016; Raja et al., 2020). Pain coping strategies can play an important role in pain management (Esteve et al., 2007; Osborne et al., 2007; López-Martínez et al., 2008; Ferreira-Valente et al., 2011, 2014, Ferreira-Valente et al., 2020; Alschuler and Otis, 2012; Jensen and Turk, 2014; Thong et al., 2017). These strategies can be categorized into adaptive coping strategies, which result in better outcomes over time, or maladaptive coping strategies that may lead to worse results over time (Haythornthwaite et al., 1998; Tan et al., 2011; Riddle et al., 2018). Some factors may directly affect pain coping strategies in CPPs such as keeping busy, while others, including environmental and social resources, may indirectly impact pain coping strategies in CPPs (Romano et al., 2011; McCluskey et al., 2015). Social resources, including social networks and perceived support from significant others, can inhibit avoidance of physical and social activities resulting in a positive influence on functional disability and long-term chronic pain outcomes (Cohen and Wills, 1985; Uchino et al., 1996; Keefe et al., 2002).

One of the main social resources for CPPs is their family caregivers. It is conceivable that family caregivers’ attitudes and beliefs about a patient’s pain can influence pain coping strategies in CPPs and make an important contribution to pain management in CPPs (Block and Boyer, 1984; Burman and Margolin, 1992; Burns et al., 1996; Cano et al., 2000; Bigatti and Cronan, 2002; Leonard et al., 2006; Broderick et al., 2011; Ferreira-Valente et al., 2011, 2014; Jensen and Turk, 2014; Taylor et al., 2017; Thong et al., 2017; Riddle et al., 2018). Pain-related attitudes can impact a person’s feelings toward pain while beliefs may influence pain-related behavior through the information that an individual considers relevant (Ajzen and Fishbein, 1977; Tait and Chibnall, 1997; Riddle et al., 2018). Perceived social support from significant others was found to play an important role as a coping strategy in CPPs (Evers et al., 2003). This support leads to adaptive pain coping and less activity avoidance, resulting in the prevention of long-term functional disability and pain (Evers et al., 2003). This is in line with the fear-avoidance models, which posit that pain-related avoidance factors including activity avoidance and pain catastrophizing are associated with the worst prognosis in CPPs (Linton, 2000; Vlaeyen and Linton, 2000). Whereas social resources (e.g., adaptive attitudes and beliefs about a patient’s pain) can help patients modify pain-related avoidance factors, potentially leading to a positive impact on adaptive pain coping strategies in CPPs.

The family caregivers’ pain attitudes-beliefs and the patients’ coping strategies toward pain might both be influenced by emotion regulation strategies of patients, but also by their caregivers (Gross, 2002; Koechlin et al., 2018). Emotions form the way we communicate with the world around us. Our emotions sometimes operate proficiently and at other times may lead us astray. Our efforts to influence our emotions in order to increase the chance that they will be helpful rather than harmful is called emotion regulation (Gross, 2015). In fact, emotion regulation has been defined as a person’s ability to regulate his/her emotional state and expression including identifying emotions, recognizing the context and situation that triggered those emotions, and adjusting responses to them (Gross, 2002, 2014; Adrian et al., 2011). Therefore, emotion regulation involves cognitive, behavioral, and psychophysiological responses to an event or stressor (Koechlin et al., 2018) and choosing different emotion regulation strategies can have different outcomes on how a person feels, thinks, and acts (Gross, 2015).

Emotion regulation has been the focus of several studies, particularly related to stress, coping, and pain (Koechlin et al., 2018). Findings supported the association between the emotion regulation strategies used and the pain experience (for a review see Koechlin et al., 2018). Suppression and acceptance (two primary forms of emotion regulation strategies) on physiological and behavioral responses during the presentation of painful stimuli, while decreasing pain and anxiety compared to the control group (Braams et al., 2012). Effective emotion regulation in family caregivers may also lead to better communication and more support between patients and caregivers (Fruzzetti and Iverson, 2006; Keefe et al., 2006; Gottman, 2013). In fact, the social context of pain, including the effect of caregivers’ responses on the patient’s pain, is critical as it may inform us about the pain source as well as the preference for pain coping strategies (Davis et al., 2015). Accordingly, the pain communication model suggested that, when patients send pain messages, these messages will be perceived by family caregivers *via* their cognitive and affective processes (Cano et al., 2005; Hadjistavropoulos et al., 2011). Subsequently, the type of response that family caregivers provide depends on these cognitive and affective processes that in turn can impact the patient’s pain experience (Metalsky et al., 1987; Raichle et al., 2011; Lemieux et al., 2013; Davis et al., 2015). For example, a brief validation training session for spouses could modify validating responses (e.g., conveying respect and acceptance) and reduced the amount of invalidating responses toward patients, which in turn had a positive effect on the CPPs’ emotions (Leong et al., 2011).

In order to tailor more effective interventions and minimize the burden of chronic pain, it is critical to identify the interaction and contribution of psychosocial factors, including pain coping, pain attitudes-beliefs, and emotion regulation strategies in both CPPs and family caregivers. To our knowledge, no previous studies consider the mediating role of emotion regulation in both CPPs and their family caregivers simultaneously as an effective interpersonal process which may change the patients’ pain experience. Therefore, the present study aimed to investigate the association between pain attitudes-beliefs in family caregivers and pain coping strategies in CPPs. We also aimed to assess whether emotional regulation in both CPPs and family caregivers would mediate the association between the caregivers’ pain attitudes-beliefs and the patients’ pain coping strategies. Specifically, we hypothesized that the family caregivers’ pain attitudes-beliefs contribute to emotion regulation in CPPs, which in turn, contributes to pain coping strategies in CPPs. Moreover, the family caregivers’ beliefs may affect their own emotional regulation, which in turn, would influence the patients’ pain coping strategies.

## 2. Materials and methods

### 2.1. Participants and procedure

Participants were recruited from four physiotherapy clinics in Isfahan, Iran. This study included 200 eligible chronic musculoskeletal pain patients (143 women and 57 men; range 15–82 years old; mean ± SD: 44.67 ± 13.8) and 200 family caregivers (a carer who lives with the patient; 143 women and 57 men; range 14–78 years old; mean ± SD: 37.04 ± 13.3; see Table 1).

**Table 1:** Demographic information.

Demographic information	Age (SD)	Female %
Chronic patients (*N* = 200)	44.67 (13.8)	71.5% (*n* = 143)
Family Caregivers (*N* = 200)	37.04 (13.3)	71.5% (*n* = 143)
Family Caregivers’ relationship with the patient	Frequency (%)	
Spouse	93 (46.5%)	
Daughter	70 (35%)	
Son	0‥7 (3.5%)	
Mother	20 (10%)	
Father	0 (0%)	
Sister	6 (3%)	
Other	4 (2%)	
Education patients	Frequency (%)	
Primary school	71 (35.5%)	
Secondary school	102 (51%)	
College	24 (12%)	
Graduate/professional school	3 (1.5%)	
Education family caregivers	Frequency (%)	
Primary school	24 (12%)	
Secondary school	122 (61%)	
College	45 (22.5%)	
Graduate/professional school	9 (4.5%)	

*Values are mean and SD or %*.

Nurses and front desk staff at these four physiotherapy clinics identified the eligible patients and notified the researcher in the field (FA) who invited patients to participate in the study. The researcher explained the study and assessed the patients for eligibility. Inclusion criteria for CPPs contained constant pain for more than 3 months, fluency in reading and writing in Farsi, being able to independently complete study questionnaires, and having at least one of the family members living with the CPPs as a caregiver. Family caregivers should be family members living with the patient and can provide most of the care and attention to the patient while not belonging to any formal network of carers. Family caregivers were identified through the question: are you the family member who is undertaking most of the care for the patient at home? (del Mar García-Calvente et al., 2004; Ojeda et al., 2014). Inclusion criteria for family caregivers included being fluent in reading and writing in Farsi and being able to independently complete study questionnaires. Exclusion criteria for CPPs and family caregivers included a medical history of major psychiatric disorder, concussion or head injury, and current drug and/or alcohol abuse.

After an initial screening for the inclusion and exclusion criteria, eligible CPPs and family caregivers signed the consent forms. Afterwards, the researcher asked questions about the patient’s pain experience, pain duration, and the family caregivers’ demographic data, and finally explained each questionnaire and provided instructions separately to both patients and their family caregivers at the physiotherapy clinics. Then, CPPs and family caregivers completed the battery of questionnaires. CPPs completed the Emotion Regulation Questionnaire (ERQ) and Coping Strategies’ Questionnaire (CSQ) along with demographic information, while family caregivers completed ERQ and Survey of Pain Attitudes (SOPA-R). CPPs answered the questionnaires independent of their family caregivers.

### 2.2. Outcome measurements

#### 2.2.1 Demographic characteristics

CPPs reported their age, sex, the level of education. Family caregivers reported age, sex, level of education, and their relationship with the patient (see Table 1).

#### 2.2.2 Pain characteristics

CPPs were referred by their medical doctors to those physiotherapy clinics due to their pain. The researcher interviewed patients in the clinics and asked each patient to report their pain intensity at the moment by using a Visual Analogue Scale (VAS) ranging from 0 (“no pain”) to 100 (“worse pain imaginable”; VAS 1) as well as rating their pain intensity in the last week (VAS 2). CPPs also reported their pain locations, type of pain experienced, treatment and medication history, diagnostic measures that have been taken, current use of analgesic medication, and pain duration. If they had constant musculoskeletal pain for at least 3 months, they were eligible to participate in the study and complete the questionnaires (see Table 2).

**Table 2:** Pain characteristics.

Pain characteristics (*N* = 200)	Mean (SD)
Pain intensity (VAS 1)	57.50 (27.45)
Pain intensity (VAS 2)	63.87 (27.47)
Pain duration (Month)	61.21 (72.9)
Pain locations	Frequency (%)
Neck	4 (2%)
Shoulder	9 (4.5%)
Spine	0 (0%)
Hand	13 (6.5%)
Knee	32 (16%)
Lower back	12 (6%)
Leg	16 (8%)
Pain in two locations	15 (7.5%)
Pain in more than two locations	99 (49.5%)
Type of pain	Frequency (%)
Burns	10 (5%)
Pressure	18 (9%)
Prickly	3 (1.5%)
Pulsing	0 (0%)
Squeezing	76 (38%)
2 types of pain	67 (33.5%)
3 types of pain or more	26 (13%)
Treatment and medication history	Frequency (%)
Medicine	0 (0%)
Physiotherapy	22 (11.5%)
Surgery	1 (0.5%)
Acupuncture	0 (0%)
Message	0 (0%)
Both medicine and physiotherapy	101 (50.5%)
2 types of therapy except medicine and physiotherapy	11 (5.5%)
3 types of therapy or more	65 (32.5%)
Diagnostic measures	Frequency (%)
Electromyography	13 (6.5%)
CT SCAN	3 (1.5%)
Radiology	27 (13.5%)
MRI	79 (39.5%)
None of them	3 (1.5%)
2 types of diagnoses	55 (27.5%)
3 types of diagnoses or more	20 (10%)
Usage of analgesic medicines during the research	Frequency (%)
Yes	123 (61.5%)
No	77 (38.5%)

#### 2.2.3. Questionnaires

Attitudes and beliefs are known to mediate influence on behavior (Fishbein and Ajzen, 1977), and the SOPA is one of the most reliable tools that allow us to examine a range of attitudes influencing pain behavior (Jensen et al., 1987), including pain control, disability, cures, medications, and solicitude of others. Cognitive factors may facilitate or inhibit adaptive functioning in the presence of pain, including coping strategies. These strategies have long been investigated in their role to identify interindividual differences in CPPs (Rosenstiel and Keefe, 1983). The items used in the CSQ seem to reflect the most common coping strategies identified by patients, researchers, and clinicians alike. Finally, individual differences in emotion regulation were shown to be important for adaptation, in the present study, to chronic pain. The ERQ measures two of the most important regulation strategies with high reliability and validity: cognitive reappraisal and expressive suppression (Gross, 2002).

#### 2.2.4. Survey of pain attitudes (SOPA, Pain Attitudes-Beliefs)

The present study used the brief version of SOPA that includes 35 items with seven subscales: (1) pain control (e.g., the amount of pain I feel is out of my control); (2) pain-related disability (e.g., if my pain continues at its present level, I will be unable to work); (3) harm (e.g., the pain I feel is a symptom that shows the damage is being done); (4) emotion (e.g., anxiety increase the pain I feel); (5) medication (e.g., medicine is one of the best treatments for chronic pain); (6) solicitude (e.g., when I hurt, I want my family to treat me better); and (7) medical cure (e.g., I pay doctors so that they will cure my pain; Jensen et al., 2000; Cano et al., 2009). Each item is assessed on a 5-point Likert scale ranging from 0 (i.e., this is completely false for me) to 4 (i.e., this is completely true for me; Jensen et al., 2000). Only family caregivers filled the SOPA questionnaire.

The SOPA has good psychometric properties (Jensen et al., 2000; Cano et al., 2009). Jensen et al. (2000) reported satisfactory internal consistency for the control subscale (α = 0.78), the disability subscale (α = 0.70), the harm subscale (α = 0.66), the emotion subscale (*a* = 0.81), the medication subscale (α = 0.78), the solicitude subscale (α = 0.81) and the medical cure subscale (α = 0.74) by using this survey on both patients. In the present sample, Cronbach’s alpha for the SOPA’s subscales of control, disability, harm, emotion, medication, solicitude, and medical cure respectively were 0.42, 0.42, 0.36, 0.66, 0.69, 0.86, 0.38. However, in the current study, all items for Cronbach’s alpha were assessed for family caregivers (α = 0.88). For the present study, we used the version hired by Khahi and colleagues as a part of a larger survey, which demonstrated an acceptable internal consistency (0.70) and test-retest reliability (0.80; Panah Khahi et al., 2012). Cronbach’s alpha of the total score was the maximum of all Cronbach’s alpha of every subscale; thus, internal consistency was considered acceptable.

#### 2.2.5. Coping strategies questionnaire

The Coping Strategies Questionnaire (CSQ) is a 44-item self-report scale that assesses coping strategies in CPPs (Rosenstiel and Keefe, 1983). It contains seven subscales. The CSQ assesses six cognitive coping strategies: diverting attention (e.g., “I try to think of something pleasant”); reinterpreting pain sensations (e.g., “I just think of it as some other sensation, such as numbness”); catastrophizing (“I worry all the time about whether it will end”); ignoring the pain sensations (e.g., “I tell myself it doesn’t hurt”); praying or hoping (e.g., “I pray that maybe it won’t last long”); coping self-statements (e.g., “I tell myself to be brave and carry it on despite the pain”) and one behavioral coping strategy: increasing the activity level (e.g., “I participate in activities such as chores or household projects”; Rosenstiel and Keefe, 1983). Moreover, the CSQ has two items about the effectiveness of coping strategies: control over pain and the ability to decrease the pain (Rosenstiel and Keefe, 1983). Each item was rated using a 7-point Likert scale (i.e., 0 = never, 6 = always; Rosenstiel and Keefe, 1983). Studies showed that the CSQ has good psychometric properties (Rosenstiel and Keefe, 1983; Williams and Keefe, 1991; Asghari and Nicholas, 2004).

Asghari and Nicholas (2004) reported satisfactory internal consistency of α = 0.72 for the diverting attention’s subscale, α = 0.82 for reinterpreting pain sensations, α = 0.75 for the catastrophizing subscale, α = 0.73 for the coping self-statements subscale, α = 0.84 for the ignoring pain sensations subscale, α = 0.60 for praying and hoping and α = 0.68 for the increasing behavioral activity subscale by using this questionnaire on a patients’ sample (Asghari and Nicholas, 2004). In the present sample, Cronbach’s alpha for diverting attention’s subscale, reinterpreting pain sensations, catastrophizing, ignoring pain sensations, praying or hoping, coping self-statements, and increasing activity level were *α* = 0.80, 0.80, 0.82, 0.85, 0.70, 0.70, 0.75, respectively. Only CPPs were asked to complete the CSQ.

#### 2.2.6. Emotion regulation questionnaire

The Emotion Regulation Questionnaire (ERQ) is a 10-item self-report scale developed by Gross and John (2003), to measure the habitual use of reappraisal and suppression. The ERQ assesses individual differences in the typical use of two emotion regulation strategies: cognitive reappraisal with six items (e.g., “I control my emotions by changing my way of thinking about my current situation”) as well as expressive suppression with four items (e.g., “I keep my emotions to myself”; Gross and John, 2003). Each item is rated on a 7-point Likert scale (1 = strongly disagree; 7 = strongly agree). The ERQ had good psychometric properties (Gross and John, 2003; Ehring et al., 2010). The present study utilized the version validated by Hasani (2016), which showed reliability of the same two factor model with satisfactory levels of internal consistency (0.81–0.91) and high test-retest reliability (0.51–0.77). Both CPPs and family caregivers were asked to complete the ERQ. The Cronbach’s alpha of cognitive reappraisal and expressive suppression were 0.74, 0.62 for CPPs, and 0.63 and 0.64 respectively for family caregivers.

### 2.3. Data analysis

This study was a cross-sectional study, in which data was collected from chronic pain patients and their family caregivers at a single point. To examine the hypothetical models of the study, the structural equation modeling (Avery et al., 2014) method was performed using AMOS 20.0 (Arbuckle, 2011). SEM can provide fit indices to investigate the predicted relationships between variables of the model (Tabachnick and Fidell, 2018). SEM is a combination of multiple regression and confirmatory factor analysis (Tabachnick and Fidell, 2018), which permits the relationship among multiple dependent or outcome variables to be examined simultaneously. The maximum likelihood was used to assess model fit. According to Byrne, several fit indices were used for parameter estimation (Taris, 2002). In the current study, the model fit is assessed using the following goodness of fit indices: (1) Chi-square (*x^2^*) is the most common index of model fit. This index is very sensitive to the sample size (especially large samples) and non-normality of the data with a non-significant χ^2^ implying goodness of fit of the model to the data (Marsh et al., 1988). (2) RMSEA is a fit measure according to population error of approximation with an RMSEA value below 0.08, demonstrating a close fit and values below 0.10, indicating reasonable errors of approximation in the population (Browne and Cudeck, 1993). (3) CFI, which is an incremental fit index and demonstrates adequate improvement in the model, was fitted by comparing the target model with a baseline model (Bentler, 1990). (4) Normed fit index (NFI; Jöreskog and Sörbom, 1986). (5) Consistent Akaike Information Criterion (CAIC; Anderson et al., 1998) and Tucker-Lewis Index (TLI; Tucker and Lewis, 1973). For the purpose of the current study, goodness of fit was evaluated using the following statistics: NFI > 0.90, CFI > 0.90, normal chi-square (3 < *x*^2^/df < 2), RMSEA and its 90% confidence interval (<0.08; Cole, 1987).

## 3. Results

### 3.1. Descriptive characteristics

Table 1 presents the demographic characteristics of CPPs and family caregivers and Table 2 demonstrates the characteristics of CPPs. Besides, Table 3 shows the psychometric properties of measures used in the study.

**Table 3:** Psychometric properties of measures used in the study.

	Variable	Indicators	N.items	α	Mean (SD)
Patients					
	ERQ1	Emotion regulation (cognitive reappraisal)	6	0.74	29.1 (8.06)
	ERQ2	Emotion regulation (expressive suppression)	4	0.62	15.12 (6.03)
	CSQ1	Pain adjustment (diverting attention)	6	0.80	16.19 (8.15)
	CSQ2	Pain adjustment (reinterpreting pain sensations)	6	0.80	12.79 (7.64)
	CSQ3	Pain adjustment (catastrophizing)	6	0.82	16.48 (8.65)
	CSQ4	Pain adjustment (ignoring pain sensations)	6	0.85	15.22 (8.44)
	CSQ5	Pain adjustment (praying or hoping)	6	0.70	27.47 (5.91)
	CSQ6	Pain adjustment (coping self-statements)	6	0.70	21.56 (6.84)
	CSQ7	Pain adjustment (increasing activity level)	6	0.75	16.07 (7.62)
Family Caregivers					
	ERQ1	Emotion regulation (cognitive reappraisal)	6	0.63	30.81 (7.01)
	ERQ2	Emotion regulation (expressive suppression)	4	0.64	14.6 (5.97)
	SOPA1	Pain attitude (pain control)	5	0.42	11.8 (4.74)
	SOPA2	Pain attitude (disability)	5	0.42	7.54 (3.9)
	SOPA3	Pain attitude (harm)	5	0.36	9.03 (3.77)
	SOPA4	Pain attitude (emotion)	5	0.66	12.77 (4.62)
	SOPA5	Pain attitude (medication)	5	0.69	11.28 (4.9)
	SOPA6	Pain attitude (solicitude)	5	0.86	14.2 (5.27)
	SOPA7	Pain attitude (medical cure)	5	0.38	12.75 (3.77)

*Values are mean and SD. Note. ERQ, emotion regulation questionnaire; CSQ, coping strategies questionnaire; SOPA, survey of pain attitudes; N. item, number of items*.

### 3.2. Preliminary analyses

**Figure 1:**
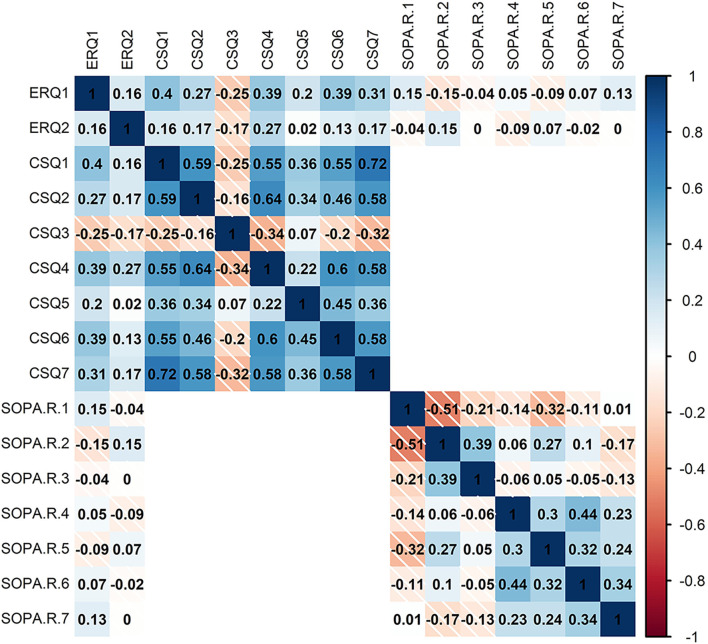
Heatmap of Pearson correlations (−1:1 shown by color legend) for SOPA, survey of pain attitude; ERQ, emotion regulation questionnaire; and CSQ, coping strategies questionnaire.

The data were examined for skewness and kurtosis. All variables were normally distributed and did not contravene the underlying assumptions for the analysis. Correlations are reported in Figure 1. Variance inflation factors (VIFs) were examined in order to check the statistical multicollinearity (all VIFs were found to be less than 5; O’brien, 2007).

### 3.3. Test of models

#### 3.3.1. Hypothesis 1

**Figure 2:**
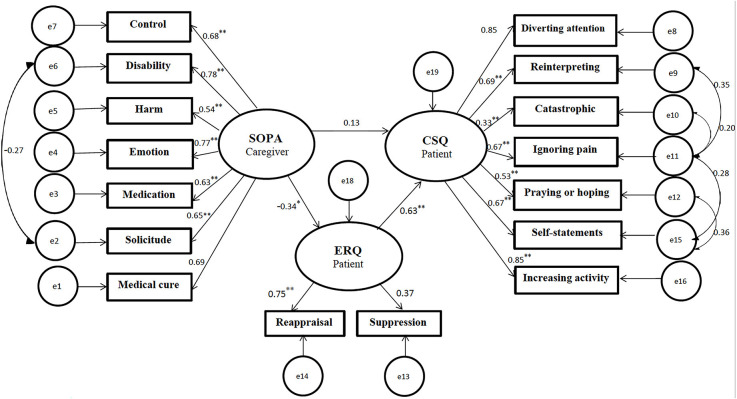
Predicted relationship based on Model 1 (with resulting standardized regression weights). All coefficients are significant (*p* < 0.05* *p* < 0.001**). SOPA, survey of pain attitudes; CSQ, coping strategies questionnaire; ERQ, emotion regulation questionnaire.

In the first model, we proposed that the family caregivers’ pain attitudes-beliefs predict pain coping strategies in CPPs (Cano et al., 2009). This model is based on cognitive-behavioral theories of chronic pain, which propose that family caregivers’ pain attitudes-beliefs impact CPPs’ responses (Fordyce et al., 1968a; Turk et al., 1983). However, cognitive-behavioral theories of chronic pain did not consider the role of emotion regulation. Our first model suggested that caregivers’ pain attitudes-beliefs indirectly predict patients’ pain coping strategies through patients’ emotion regulation (see Figure 2). It also suggested that caregivers’ pain attitudes-beliefs predict emotion regulation of patients. Modification indices suggested that the correlation of the residuals of CSQ2 and CSQ4, CSQ3 and CSQ4, CSQ4 and CSQ6, CSQ5 and CSQ6, SOPA2 and SOPA6 would increase the fitness of the model. These modifications would not violate the theoretical basis of the model (Hoyle, 1995; MacCallum and Austin, 2000). After modification, the goodness of fit statistics of this model indicated an acceptable fit (*x^2^* = 116.38 (96) = 1.21, *p* < 0.05, NFI = 0.91, CAIC = 368.31, TLI = 0.97, CFI = 0.98 and RMSEA = 0.03; see Table 4).

**Table 4:** Goodness of fit indices for two tested models.

Model	NFI	CAIC	RMSEA	RMR	IFI	CFI	TLI	*χ* ^2^	df	df/*χ*^2^	Δ*χ^2^*
M1	0.91	368.31	0.03	2.04	0.98	0.98	0.97	116.38	96	1.21	4.13
M2	0.90	366.15	0.03	1.77	0.97	0.97	0.97	120.51	97	1.24	56.65

*Note. Δ/χ^2^, the difference between two competitive models; NFI, normed fit index; CAIC, calculated consistent akaike information criterion; RMSEA, root-mean-square-error of approximation; IFI, incremental fit index; CFI, comparative fit index; TLI, Tucker-Lewis Index*.

#### 3.3.2. Hypothesis 2

**Figure 3:**
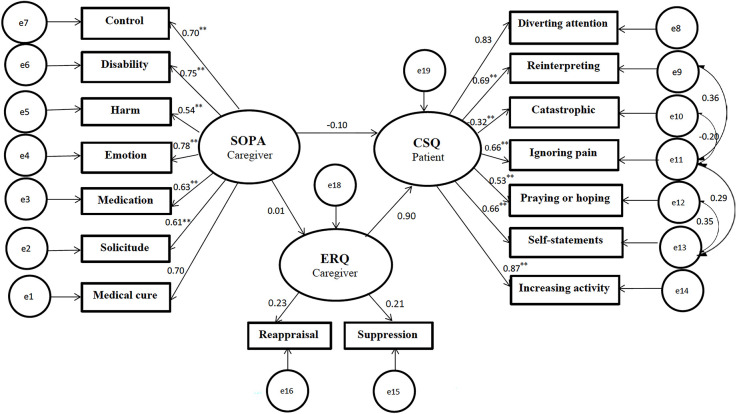
Predicted relationship based on Model 2 (with resulting standardized regression weights). All coefficients are significant (*p* < 0.001**). SOPA, survey of pain attitudes; CSQ, coping strategies questionnaire; ERQ, emotion regulation questionnaire.

We also examined an alternative model that considered the mediating role of the family caregivers’ emotion regulation in the relationship between their pain attitudes-beliefs and pain coping strategies in CPPs (see Figure 3). According to this model, the caregivers’ pain attitudes-beliefs indirectly predict the patient’s pain coping strategies through the caregivers’ emotion regulation. Furthermore, this model proposed that the caregivers’ pain attitudes-beliefs directly predict their emotion regulation. Modification indices offered the correlation of the residuals of CSQ2 and CSQ4, CSQ3 and CSQ4, CSQ4 and CSQ6, CSQ5 and CSQ6. After modification, the fit indices for the second model indicated an acceptable fit (*x^2^* = 120.51 (97) = 1.24, *p* < 0.05, NFI = 0.90, CAIC = 366.15, TLI = 0.97, CFI = 0.97 and RMSEA = 0.03; see Table 4). To re-evaluate the final model bootstrapping method with 5,000-resample generation and 95% interval confidence was conducted to correct possible biases (Preacher and Hayes, 2004).

## 4. Discussion

The current study investigated the role of emotion regulation in the relationship between family caregivers’ pain attitudes-beliefs and CPPs’ coping strategies. To the best of our knowledge, there has been no research to investigate the mediating role of emotion regulation in both CPPs and their family caregivers simultaneously as an effective interpersonal process that can change the pain experience in this relationship. Our results showed that family caregivers’ pain attitudes-beliefs correlate with pain coping strategies in patients with chronic musculoskeletal pain, and this relationship is mediated by emotion regulation strategies of both CPPs and family caregivers. These findings suggest that emotion regulation strategies may alter the family caregivers’ pain attitudes-beliefs (toward the patient), as well as the choice of pain coping strategies in the patient (Fordyce et al., 1968b; Geisser et al., 1999; Paquet et al., 2005; Leong et al., 2011; Tan et al., 2011; Braams et al., 2012; Prenevost and Reme, 2017; Koechlin et al., 2018; Riddle et al., 2018). Therefore, providing emotion regulation training for both patients and their family caregivers can be considered when developing treatment programs for CPPs (Hamilton et al., 2004; Paquet et al., 2005; Connelly et al., 2007; Agar-Wilson and Jackson, 2012; Braams et al., 2012; Compas et al., 2017; Aaron et al., 2020).

The current study was focused on the interaction between CPPs and their family caregivers as they play an important role in the pain experience. Our findings revealed an association between pain attitudes-beliefs in family caregivers and pain coping strategies in patients. This finding is consistent with the cognitive-behavioral theory, proposing that the development, maintenance, and management of pain in CPPs is probably affected by their family caregivers’ pain attitudes-beliefs and responses (Metalsky et al., 1987; Cano et al., 2009; Raichle et al., 2011; Lemieux et al., 2013; Davis et al., 2015). Besides, behavioral evidence also supports that partners’ pain attitudes-beliefs significantly correlated with patients’ pain severity and other indicators of pain coping strategies (Cano et al., 2009). However, this study considered partners’ pain attitudes-beliefs separately, some subscales, such as disability, emotion, control, and medication, were significantly correlated with partners’ indicators of pain adjustment, while some partners’ pain attitudes-beliefs subscales were not (Cano et al., 2009). In line with our results, evidence supported that family caregivers’ pain attitudes-beliefs make an important contribution to pain management in CPPs by affecting patients’ coping strategies (Costa et al., 2011; Ferreira-Valente et al., 2011, 2014, Ferreira-Valente et al., 2020; Jensen and Turk, 2014; Thong et al., 2017).

Another key factor that can influence the pain experience are emotion regulation skills (Gross, 2002; Hamilton et al., 2004; Paquet et al., 2005; Connelly et al., 2007; Agar-Wilson and Jackson, 2012; Braams et al., 2012; Compas et al., 2017; Koechlin et al., 2018; Aaron et al., 2020). Previous studies suggest that chronic pain is associated with various psychological difficulties (e.g., anxiety, depression, fear, and anger) as well as several emotion regulation difficulties in CPPs that contribute to overall dysfunction (for a review see Romano and Turner, 1985; Rudy et al., 1988; Brown, 1990; Fishbain et al., 1997; Asmundson and Katz, 2009; Sheng et al., 2017; Koechlin et al., 2018). Additionally, pain is affected by the broader social context, including the patients’ relationship with their family caregivers (Romano et al., 2011; Davis et al., 2015; McCluskey et al., 2015). Therefore, adaptive and maladaptive emotion regulation strategies of family caregivers can also influence the pain experience in CPPs. The results of our study support an association between family caregivers’ pain attitudes-beliefs and patients’ coping strategies, which is mediated by the patients’ emotion regulation. This finding is consistent with the results of a cross-sectional study that examined the association between emotion regulation skills and pain adjustment in CPPs (Agar-Wilson and Jackson, 2012). Efficacy in patients’ emotion regulation was associated with increased quality of life and decreased negative emotions, even after controlling for the effect of other factors, including adjustment, pain coping efficacy, and pain coping (Agar-Wilson and Jackson, 2012). These results suggest that understanding emotion regulation strategies in CPPs may lead to better recognition of individual variability in pain adjustment (Agar-Wilson and Jackson, 2012).

We also found that the emotion regulation of family caregivers contributes to the relationship between the family caregivers’ pain attitudes-beliefs and patients’ coping strategies. This finding is consistent with the pain communication model, which suggested that, when CPPs send pain messages, these will be perceived by the family caregiver, and the family caregivers’ responses are determined by their cognitions and affective processes which, in turn, can affect the patients’ pain experience (Metalsky et al., 1987; Raichle et al., 2011; Lemieux et al., 2013; Davis et al., 2015). Studies supported that effective emotion regulation in family caregivers leads to better communication and enhanced support between CPPs and family caregivers (Keefe et al., 2006). For example, validating responses (i.e., empathic responses that validate the other person’s experience) may improve adaptive emotion regulation in couples and help CPPs to process pain (Leong et al., 2011). A study found that satisfaction with spouses’ responses reduced overwhelmed and helpless feelings in CPPs during their daily pain (Leong, 2010). This suggests that validating responses from partners may improve emotion regulation skills and lead to pain reduction in patients (Leong, 2010). In parallel, the results of another study showed that acceptance and suppression (two distinct emotion regulation strategies) from spouses led to pain reduction in chronic pain partners. However, invalidating responses from spouses (e.g., ignoring the partner’s emotion, rejecting and disregarding another person) interrupt emotion regulation and lead to an enhanced pain experience in chronic pain partners (Leong et al., 2011). Therefore, validating responses may lead to the use of adaptive pain coping strategies by improving emotion regulation skills, whereas invalidating responses may disturb adaptive pain coping strategies (Leong et al., 2011; Prenevost and Reme, 2017; Riddle et al., 2018). Family caregivers’ responses affect a patient’s choice of coping strategies and the efficacy of specific coping strategies (Davis et al., 2015). This association can be modulated by the emotion regulation skills of both CPPs and their family caregivers.

In conclusion, results from the current study showed that the emotion regulation of CPPs and family caregivers may mediate the association between caregivers’ pain attitudes-beliefs and the use of pain coping strategies in CPPs. Adaptive emotion regulation skills in both patients and their family caregivers may contribute to better pain management in patients. These findings are clinically relevant with immediate clinical applications, given that emotional regulation is a modifiable mechanism that could be targeted by interventions. When it comes to pain management and developing interventions for CPPs, it is important to educate patients and their family caregivers to better understand the impact of adaptive and maladaptive emotion regulation skills and their association with pain coping, and to help patients to have a better understanding of how these skills can impact their behaviors, increasing the level of control over their pain and pain coping strategies. Future studies should consider developing non-pharmacological interventions focused on improving emotion regulation skills in both CPPs with different types of chronic pain and family caregivers in the early stages of acute pain, which may help prevent pain from becoming chronic.

The current results must be interpreted in view of a number of limitations. Firstly, due to the cross-sectional design of the study, path directions are theoretical, and causality cannot be inferred. Studies employing a longitudinal design are needed. Secondly, measures often have similar items and in the case of some measures, for example, emotion regulation and coping strategies, there should be conceptual overlap. In these cases, the overlap between the residuals is not surprising. Thirdly, we considered emotion regulation generally in the current study. Future studies could consider different emotional regulation strategies separately that should be more exact. Fourthly, only patients with chronic musculoskeletal pain and their family caregivers were recruited for this study, which might limit the generalizability of the findings to patients with other types of pain (e.g., cancer pain). Finally, some factors, such as relationship quality, which can play an important role in understanding how emotion regulation plays a mediating role in the relationship between family caregivers’ beliefs and coping strategies, have not been investigated in this study.

## Data availability statement

The raw data supporting the conclusions of this article will be made available by the authors, without undue reservation.

## Ethics statement

The studies involving human participants were reviewed and approved and all study procedures were approved by the Ethics Committee of Shahid Beheshti University, Tehran, Iran. All participants provided written informed consent, acknowledging their right to withdraw from the experiment without prejudice. Written informed consent to participate in this study was provided by the participants’ legal guardian/next of kin.

## Author contributions

FA was involved in data acquisition, design, analysis, data interpretation, and writing. ZD was involved in analysis, data interpretation, and writing. MD and AK were involved in the design, analysis, and writing. All authors contributed to the article and approved the submitted version.
